# Identification of *CDC42BPG* as a novel susceptibility locus for hyperuricemia in a Japanese population

**DOI:** 10.1007/s00438-017-1394-1

**Published:** 2017-11-09

**Authors:** Yoshiki Yasukochi, Jun Sakuma, Ichiro Takeuchi, Kimihiko Kato, Mitsutoshi Oguri, Tetsuo Fujimaki, Hideki Horibe, Yoshiji Yamada

**Affiliations:** 1 Department of Human Functional Genomics, Advanced Science Research Promotion Center, Organization for the Promotion of Regional Innovation, Mie University, 1577 Kurima-machiya, Tsu, Mie 514–8507 Japan; 2 CREST, Japan Science and Technology Agency, Kawaguchi, Saitama Japan; 3 Computer Science Department, College of Information Science, University of Tsukuba, Tsukuba, Ibaraki Japan; 4 RIKEN Center for Advanced Intelligence Project, Chuo-ku, Tokyo Japan; 5 Department of Computer Science, Nagoya Institute of Technology, Nagoya, Aichi Japan; 6 Department of Internal Medicine, Meitoh Hospital, Nagoya, Aichi Japan; 7 Department of Cardiology, Kasugai Municipal Hospital, Kasugai, Aichi Japan; 8 Department of Cardiovascular Medicine, Inabe General Hospital, Inabe, Mie Japan; 9 Department of Cardiovascular Medicine, Gifu Prefectural Tajimi Hospital, Tajimi, Gifu Japan

**Keywords:** Chronic kidney disease, Creatinine, Estimated glomerular filtration rate, Generalized estimating equation, Hyperuricemia, Uric acid

## Abstract

Chronic kidney disease and hyperuricemia are serious global health problems. Recent genome-wide association studies have identified various genetic variants related to these disorders. However, most studies have been conducted in a cross-sectional manner. To identify novel susceptibility loci for chronic kidney disease or hyperuricemia, we performed longitudinal exome-wide association studies (EWASs), using ~ 244,000 genetic variants and clinical data of Japanese individuals who had undergone annual health checkups for several years. After establishing quality controls, the association of renal function-related traits in 5648 subjects (excluding patients with dialysis and population outliers) with 24,579 single nucleotide variants (SNVs) for three genetic models (*P* < 3.39 × 10^− 7^) was tested using generalized estimating equation models. The longitudinal EWASs revealed novel relations of five SNVs to renal function-related traits. Cross-sectional data for renal function-related traits in 7699 Japanese subjects were examined in a replication study. Among the five SNVs, rs55975541 in *CDC42BPG* was significantly (*P* < 4.90 × 10^− 4^) related to the serum concentration of uric acid in the replication cohort. We also examined the SNVs detected in our longitudinal EWASs with the information on *P* values in GKDGEN meta-analysis data. Four SNVs in *SLC15A2* were significantly associated with the estimated glomerular filtration rate in European ancestry populations, although these SNVs were related to the serum concentration of uric acid with borderline significance in our longitudinal EWASs. Our findings indicate that *CDC42BPG* may be a novel susceptibility locus for hyperuricemia.

**Electronic supplementary material:**

The online version of this article (10.1007/s00438-017-1394-1) contains supplementary material, which is available to authorized users.

## Introduction

Chronic kidney disease (CKD) and hyperuricemia are caused by renal function abnormalities and are multifactorial disorders resulting from interactions between genetic background and environmental factors. The goal of the present study was to identify novel susceptibility loci for CKD or hyperuricemia in Japanese individuals. CKD is a global health problem and an independent risk factor for cardiovascular diseases (Schiffrin et al. 2007; Hill et al. 2016; Webster et al. 2017). Recent genome-wide association studies (GWASs) have identified various genetic variants that confer susceptibility to CKD-related traits. A previous GWAS examined relations between genetic variants and the prevalence of CKD or estimated glomerular filtration rate (eGFR), based on serum creatinine and cystatin C levels in four population-based cohorts with European ancestry, and identified several genetic loci related to any of the clinical parameters examined (Köttgen et al. 2009). Several GWASs consistently identified a genetic variant at rs12917707 in the *UMOD* gene as a CKD susceptibility locus (Chambers et al. 2010; Böger et al. 2011; Pattaro et al. 2016). However, a recent GWAS for Icelanders did not detect this association (Sveinbjornsson et al. 2014). A meta-analysis of GWASs for 71,149 East Asian individuals, including those in an in silico replication study, identified another genetic variant, rs11864909, in *UMOD* and the linkage disequilibrium (LD) with rs12917707 was not strong (*r* ^2^ = 0.02), although rs12917707 was excluded from the GWAS meta-analysis because of low minor allele frequency (MAF < 0.01) (Okada et al. 2012). This analysis showed that 32 loci were significantly associated with four kidney function-related traits (blood urea nitrogen, serum creatinine, eGFRcrea, and uric acid).

Hyperuricemia is an important risk factor for gout (Lioté 2003). Overproduction of uric acid or decreased renal uric acid excretion may cause this disorder. Two meta-analyses of GWASs for population-based cohorts with European ancestry commonly identified relations between serum concentrations of uric acid and single nucleotide variants (SNVs) in six loci (*ABCG2, GCKR, PDZK1, SLC2A9, SLC17A1*, and *SLC22A11*) (Kolz et al. 2009; Yang et al. 2010). In previous studies, several solute carrier family (SLC) genes showed associations with hyperuricemia or gout in diverse ethnic groups (Matsuo et al. 2008; Köttgen et al. 2013; Phipps-Green et al. 2016; Nakayama et al. 2017). However, allele frequencies of SNVs associated with serum concentrations of uric acid varied across different ethnic groups (Köttgen et al. 2013). Therefore, disease-associated SNVs are different between ethnic groups owing to genetic background differences.

Extensive large-scale GWASs for renal function-related traits have been conducted focusing on populations with European ancestry. Therefore, it is possible that genetic relations in non-European populations have not been definitively defined. Conventional GWASs have been conducted in a cross-sectional manner that measures traits at a single point in time. In the present study, we traced longitudinal changes of anthropometric and clinical data in 5648 Japanese individuals who had undergone annual health checkups for several years. On the basis of these data, we performed longitudinal exome-wide association studies (EWASs) with the use of exome-array-based genotyping methods to explore novel susceptibility loci for CKD or hyperuricemia in the Japanese cohort.

## Methods

### Study subjects

A total of 5648 community-dwelling individuals were recruited from those who visited the Health Care Center of Inabe General Hospital (Inabe, Mie, Japan) for an annual health check-up from April 2003 to March 2014. All participants had each undergone 1–11 medical examinations, and the average follow-up period was 5 years. We refer to this cohort as the “discovery cohort.” Methods for the collection and storage of medical examination data and genomic DNA samples have been described previously (Yamada et al. 2015). Cross-sectional data for CKD- and hyperuricemia-related traits in 7699 Japanese subjects (Gifu Prefectural Tajimi Hospital, Tajimi; Gifu Prefectural General Medical Center, Gifu; Japanese Red Cross Nagoya First Hospital, Nagoya; Hirosaki University Hospital and Hirosaki Stroke Center, Hirosaki, Japan) were used for replication studies of candidate SNVs identified from our longitudinal EWASs. We refer to this cohort as the “replication cohort”.

Glomerular filtration rate was estimated with the use of the simplified prediction equation derived from the modified version of that described in the Modification of Diet in Renal Disease (MDRD) Study, as proposed by the Japanese Society of Nephrology (Zhang et al. 2017): eGFR (mL min^− 1^ 1.73 m^− 2^) = 194 × [age (years)]^−0.287^ × [serum creatinine (mg/dL)]^−1.094^ × [0.739 if female]. The National Kidney Foundation-Kidney Disease Outcomes Quality Initiative guidelines recommend a diagnosis of CKD if eGFR is < 60 mL min^− 1^ 1.73 m^− 2^ (Go et al. 2004). We thus adopted the criterion of an eGFR of < 60 mL min^− 1^ 1.73 m^− 2^ (actual range 12.2 to 59.9 mL min^− 1^ 1.73 m^− 2^) for the diagnosis of CKD. The control individuals for the EWAS of CKD had an eGFR of ≥ 90 mL min^− 1^ 1.73 m^− 2^ (actual range 90.1 to 595.5 mL min^− 1^ 1.73 m^− 2^) and did not appear to have functional or structural abnormalities of the kidneys or a history of renal disease. Subjects with hemodialysis or peritoneal dialysis were excluded from the study.

Hyperuricemia was defined by the serum uric acid concentration of > 7.0 mg/dL (> 416 µmol/L, actual range, 416.4 to 814.9 µmol/L), as proposed by the Japanese Society of Gout and Nucleic Acid Metabolism (Japanese Society of Gout and Nucleic Acid Metabolism 2012), or the taking of uric acid-lowering medication. Individuals taking drugs that may cause secondary hyperuricemia were excluded. The control individuals for the EWAS of hyperuricemia had a serum uric acid concentration of ≤ 416 µmol/L (actual range, 29.7 to 410.4 µmol/L) and had no history of hyperuricemia, gout, or uric acid-lowering medication.

**Fig. 1:**
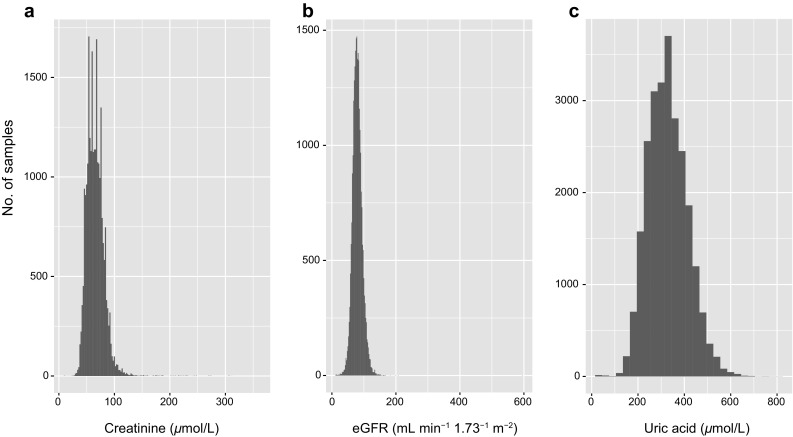
Count distributions for longitudinal data of serum concentration of creatinine (**a**), eGFR (**b**), and serum concentration of uric acid (**c**) in the discovery cohort. *eGFR* estimated glomerular filtration rate

The distributions of eGFR and serum concentrations of creatinine and uric acid are shown in Fig. 1. In the longitudinal EWASs for eGFR or the serum concentration of creatinine, a total of 5636 subjects were examined (a total of 25,371 examinations). A total of 5487 subjects were examined in the EWAS for the serum concentration of uric acid (a total of 24,873 examinations). In the EWASs for the prevalence of CKD or hyperuricemia, 1646 individuals (606 subjects with CKD and 1040 controls; a total of 7583 examinations) or 5487 individuals (847 subjects with hyperuricemia and 4640 controls; a total of 24,873 examinations) were examined, respectively. The replication cohort involved 2517 subjects with CKD and 885 controls in the study for CKD, or 972 subjects with hyperuricemia and 4584 controls in the study for hyperuricemia.

### Longitudinal EWAS

A longitudinal EWAS for the discovery cohort was performed based on ~ 244,000 genetic variants and longitudinal data of medical examinations. Infinium HumanExome-12 ver 1.2 BeadChip and Infinium Exome-24 ver 1.0 BeadChip (Illumina, San Diego, CA, USA) were used for genotyping subjects in the discovery cohort. These arrays include putative functional exonic variants selected from > 12,000 individual exome and whole-genome sequences across diverse ethnic populations (Grove et al. 2013). We performed quality controls and discarded the following SNVs: (1) monomorphic sites, (2) SNVs with MAF of < 0.05, (3) SNVs in which the genotype distribution significantly deviated from Hardy–Weinberg equilibrium (*P* < 0.001) in controls, and (4) SNVs located on mitochondrial DNA or sex chromosomes. Among Inabe subjects, four were identified as population outliers by principal component analysis (PCA) of SNVs, using the EIGENSTRAT method (Price et al. 2006) via JMP Genomics version 6.0 (SAS Institute, Cary, NC, USA) to detect population stratification on a genome-wide scale. The four outliers were removed from our longitudinal EWAS.

Next, we converted the genotyping data of the subjects into numeric data with dominant, additive, and recessive models, resulting in 24,579 SNVs. The dominant and recessive models were defined as “0, AA; 1, AB + BB” and “0, AA + AB; 1, BB” (A, major allele; B, minor allele), respectively, whereas the additive model was defined as “0, AA; 1, AB; 2, BB”. Quantile–quantile plots for the *P* values in the three genetic models are shown in Online Resources 1–3. The genomic inflation factor (*λ*) of *P* values ranged from 1.05 to 1.21.

### Statistical analyses

To examine associations between SNVs and longitudinal changes in CKD-related traits (the prevalence of CKD, eGFR, and serum concentration of creatinine), we used the generalized estimating equation (GEE) model (Liang and Zeger 1986; Hanley et al. 2003) with adjustments for age, gender, and the prevalence of hypertension and type 2 diabetes mellitus using the R package ‘geepack’ (Halekoh et al. 2006). In longitudinal EWASs for hyperuricemia-related traits (the prevalence of hyperuricemia and serum concentration of uric acid), the association with genetic variants was tested using the GEE model with adjustments for age and gender. The wave argument was used to specify the ordering of repeated measurements within individuals. The statistical significance of the association was *P* < 3.39 × 10^− 7^ (0.05/24,579 SNVs × 6) for each genetic model, after Bonferroni’s correction to compensate for multiple comparisons of genotypes with the clinical parameters. Sitlani et al. (Sitlani et al. 2015) reported that a small effective sample size can increase the chances of generating type I errors. They recommended the use of ‘approxdf’, a scale of small effective sample size, and an approxdf of ≥ 10 could reduce type I errors. Therefore, we estimated the approxdf by the R package ‘bosswithdf’ (Voorman et al. 2012; Sitlani et al. 2015). To solve the issue of association with small effective sample sizes, we set to strict approxdf threshold, and discarded SNVs with approxdf of < 30.

The association of candidate SNVs identified from our longitudinal EWASs was tested in the replication cohort using the Chi-square test (for categorical data) or linear regression analysis (for quantitative data). In addition, we also examined relations of the candidate SNVs identified, using information on *P* values in datasets of four meta-analysis studies (Köttgen et al. 2010; Pattaro et al. 2016; Gorski et al. 2017; Li et al. 2017) from GKDGEN meta-analysis data which are publicly available (https://www.nhlbi.nih.gov/research/intramural/researchers/ckdgen).

### Estimates of linkage disequilibrium

We estimated the LD among SNVs, using Haploview version 4.2 program (Barrett et al. 2005). In addition, we surveyed the LD between a candidate SNV detected from our longitudinal EWASs and other SNVs that were not included in the exome arrays used, employing LDlink web-based tools [https://analysistools.nci.nih.gov/LDlink/, (Machiela and Chanock 2015)]. The regulatory potential of each SNV was based on RegulomeDB scores [http://www.regulomedb.org/, (Boyle et al. 2012)]. Relations of candidate SNVs to disorders reported in previous studies were investigated using DisGeNET [http://www.disgenet.org/web/DisGeNET/, (Piñero et al. 2015)], GWAS Catalogue [https://www.ebi.ac.uk/gwas/, (MacArthur et al. 2017)], and GWAS Central [http://www.gwascentral.org/, (Beck et al. 2014)] databases.

## Results

### Characteristics of subjects in the discovery cohort

Characteristics of 5648 subjects in the discovery cohort, including 606 subjects with CKD and 1040 controls, and 847 subjects with hyperuricemia and 4640 controls, are shown in Table 1. The prevalence of both CKD and hyperuricemia was higher in males than in females; the proportion of males was 64.7% in subjects with CKD and 94.3% in subjects with hyperuricemia. The prevalence of hypertension, type 2 diabetes mellitus, and dyslipidemia was higher in patients with CKD or hyperuricemia than in corresponding controls. Weight, body mass index, waist circumference, systolic and diastolic blood pressures, fasting plasma glucose, blood hemoglobin A_1c_, and serum concentrations of triglycerides, low-density lipoprotein cholesterol, creatinine, and uric acid, but not height or eGFR, were also greater in patients with CKD or hyperuricemia than in corresponding controls.

**Table 1:** Characteristics of study subjects in the discovery cohort without dialysis patients or population outliers identified by the PCA method

Characteristic	Control^a^	CKD^a^	Control^a^	Hyperuricemia^a^
No. of subjects^b^	1040	606	4640	847
Sex (male/female, %)^b^	50.4/49.6	64.7/35.3	49.4/50.6	94.3/5.7
Age (years)	46.6 ± 0.14 (5608)	64.4 ± 0.21 (1975)	53.2 ± 0.08 (20,775)	52.1 ± 0.18 (4103)
Height (cm)	162.8 ± 0.12 (5581)	161.2 ± 0.21 (1923)	161.5 ± 0.06 (20,686)	168.6 ± 0.11 (4064)
Weight (kg)	60.7 ± 0.17 (5581)	61.7 ± 0.27 (1923)	59.6 ± 0.08 (20,686)	69.9 ± 0.19 (4064)
Body mass index (kg/m^2^)	22.8 ± 0.05 (5581)	23.6 ± 0.07 (1923)	22.7 ± 0.02 (20,686)	24.5 ± 0.06 (4064)
Waist circumference (cm)	80.2 ± 0.15 (4130)	83.1 ± 0.24 (1446)	80.0 ± 0.07 (16,140)	85.6 ± 0.16 (3182)
Smoking (%)	41.4 (5608)	33.0 (1975)	35.2 (20,775)	65.8 (4103)
Hypertension (%)	23.3 (5608)	63.5 (1975)	31.4 (20,775)	48.2 (4103)
Systolic blood pressure (mmHg)	118.0 ± 0.21 (5581)	126.2 ± 0.38 (1921)	119.8 ± 0.11 (20,684)	125.2 ± 0.25 (4062)
Diastolic blood pressure (mmHg)	73.1 ± 0.16 (5581)	77.0 ± 0.28 (1921)	73.9 ± 0.08 (20,684)	80.2 ± 0.19 (4062)
Type 2 diabetes mellitus (%)	10.5 (5608)	21.5 (1975)	11.6 (20,775)	12.5 (4103)
Fasting plasma glucose (mmol/L)	5.62 ± 0.018 (5601)	5.78 ± 0.028 (1957)	5.57 ± 0.008 (20,750)	5.74 ± 0.015 (4094)
Blood hemoglobin A_1c_ (%)	5.65 ± 0.012 (4000)	5.88 ± 0.015 (1711)	5.70 ± 0.005 (16,245)	5.72 ± 0.010 (2854)
Dyslipidemia (%)	49.4 (5608)	73.7 (1975)	57.5 (20,775)	74.7 (4103)
Serum triglycerides (mmol/L)	1.23 ± 0.015 (5599)	1.40 ± 0.020 (1967)	1.18 ± 0.005 (20,760)	1.73 ± 0.020 (4100)
Serum HDL-cholesterol (mmol/L)	1.62 ± 0.006 (5598)	1.52 ± 0.010 (1963)	1.65 ± 0.003 (20,757)	1.44 ± 0.006 (4098)
Serum LDL-cholesterol (mmol/L)	3.05 ± 0.011 (5393)	3.17 ± 0.018 (1854)	3.18 ± 0.005 (20,040)	3.28 ± 0.013 (3938)
Serum creatinine (µmol/L)	52.7 ± 0.12 (5608)	92.2 ± 0.58 (1975)	63.2 ± 0.10 (20,701)	78.2 ± 0.28 (4085)
eGFR (mL min^− 1^ 1.73^− 1^ m^− 2^)	100.7 ± 0.16 (5608)	52.9 ± 0.17 (1975)	80.6 ± 0.11 (20,701)	74.3 ± 0.24 (4085)
Serum uric acid (µmol/L)	303.5 ± 1.11 (5448)	374.7 ± 1.99 (1926)	300.7 ± 0.44 (20,775)	462.7 ± 0.70 (4103)

*CAD* coronary artery disease, *MI* myocardial infarction, *CI* cerebral infarction, *HDL* high-density lipoprotein, *LDL* low-density lipoprotein, *eGFR* estimated glomerular filtration rate

^a^ Values in parentheses indicate the numbers of measurements taken. Quantitative data are means and standard error

^b^ The numbers are based on data examined in the latest year

### SNVs associated with CKD and hyperuricemia in longitudinal EWASs

The GEE model with adjustments for age, gender, and the prevalence of hypertension and type 2 diabetes mellitus in the dominant and additive models showed significant relations (*P* < 3.39 × 10^− 7^) of rs11543349 in *OGFR* to serum creatinine concentration and eGFR (Table 2 and Online Resource 4). Approxdf values were 262 and 71 in the dominant and additive models, respectively. The association of rs11543349 with the serum concentration of creatinine showed the significance level with *P* = 1.2 × 10^− 8^ and 2.7 × 10^− 8^ in dominant and additive models, respectively. According to DisGeNET, GWAS Catalogue, and GWAS Central databases, the relation of this SNV to renal function has not been reported.

**Table 2:** Newly identified SNVs associated with CKD, hyperuricemia, serum concentrations of creatinine or uric acid, or eGFR in the discovery cohort

Trait/disease	RefSNP ID	Location^a^	Genotype	CKD^b^	Controls^b^	Hyperuricemia^b^	Controls^b^	Creatinine	eGFR	Uric acid
Creatinine	rs11543349	20: 62,813,587	GG	691 (35.0%)	2549 (45.5%)	1658 (40.4%)	8668 (41.7%)	75.8 ± 0.97	79.6 ± 0.17	327.1 ± 0.85
eGFR	*OGFR*		GC	969 (49.1%)	2476 (44.2%)	1932 (47.1%)	9543 (45.9%)	77.5 ± 0.92	78.1 ± 0.16	330.5 ± 0.80
			CC	315 (15.9%)	583 (10.4%)	513 (12.5%)	2564 (12.3%)	81.7 ± 2.20	76.6 ± 0.31	331.3 ± 1.56
Uric acid	rs2239709	6: 31,539,670	CC	1303 (66.0%)	3474 (61.9%)	2665 (65.0%)	13,082 (63.0%)	76.3 ± 0.76	78.4 ± 0.14	331.6 ± 0.68
	*DDX39B*		CT	621 (31.4%)	1905 (34.0%)	1326 (32.3%)	6875 (33.1%)	80.5 ± 1.30	78.6 ± 0.20	327.1 ± 0.97
			TT	51 (2.6%)	229 (4.1%)	112 (2.7%)	818 (3.9%)	66.2 ± 1.59	80.2 ± 0.48	306.2 ± 2.86
	rs2071593	6: 31,545,022	GG	1306 (66.1%)	3482 (62.1%)	2674 (65.2%)	13,104 (63.1%)	76.3 ± 0.75	78.4 ± 0.14	331.7 ± 0.68
	*NFKBIL1*		GA	618 (31.3%)	1897 (33.8%)	1319 (32.1%)	6853 (33.0%)	80.5 ± 1.31	78.6 ± 0.20	327.0 ± 0.97
			AA	51 (2.6%)	229 (4.1%)	110 (2.7%)	818 (3.9%)	66.1 ± 1.59	80.2 ± 0.48	305.8 ± 2.85
	rs55975541	11: 64,829,729	GG	1375 (69.6%)	3934 (70.1%)	2956 (72.0%)	14,152 (68.1%)	74.8 ± 0.66	78.8 ± 0.13	333.6 ± 0.65
	*CDC42BPG*		GA	553 (28.0%)	1551 (27.7%)	1058 (25.8%)	6048 (29.1%)	82.5 ± 1.53	78.1 ± 0.21	320.9 ± 1.04
			AA	47 (2.4%)	123 (2.2%)	89 (2.2%)	575 (2.8%)	86.8 ± 4.85	76.0 ± 0.68	305.6 ± 3.90
	rs12801636	11: 65,623,846	AA	509 (25.8%)	1405 (25.1%)	997 (24.3%)	5297 (25.5%)	79.4 ± 1.41	78.2 ± 0.23	324.1 ± 1.10
	*PCNX3*		AG	979 (49.6%)	2846 (50.7%)	1967 (47.9%)	10,631 (51.2%)	78.7 ± 0.95	78.5 ± 0.16	328.3 ± 0.76
			GG	487 (24.7%)	1357 (24.2%)	1139 (27.8%)	4847 (23.3%)	72.1 ± 0.99	79.0 ± 0.21	336.4 ± 1.14

*CKD* chronic kidney disease, *eGFR* estimated glomerular filtration rate, *Std.err* standard error, *MAF* minor allele frequency

^a^ Location in NCBI build GRC38

^b^ Values indicate the numbers of measurements taken, with the percentages in parentheses

**Fig. 2:**
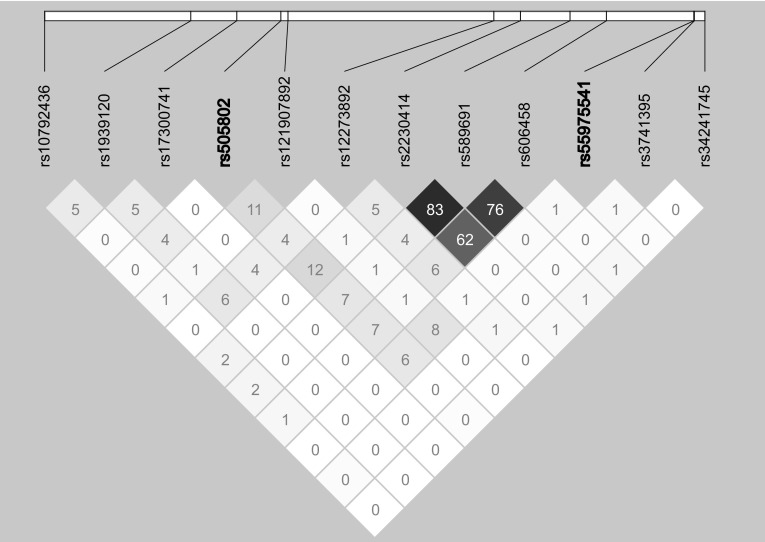
Linkage disequilibrium (LD) of 12 biallelic sites across approximately 475 kb genomic region at 11q13.1 using single nucleotide variations (SNVs) data used in the present study. The diagram was depicted by the Haploview version 4.2. The SNVs with the minor allele frequency of < 0.01 were removed from the analysis. The hyperuricemia-associated SNVs (rs505802 of *SLC22A12* and rs55975541 of *CDC42BPG*) identified by the generalized estimating equation model are shown in bold

The GEE model with adjustments for age and gender showed that 16 SNVs were significantly (*P* < 3.39 × 10^− 7^) associated with the prevalence of hyperuricemia or serum concentration of uric acid in the three genetic models, and their approxdf values were > 30 (Online Resource 4). Of these SNVs, nine are located at seven gene loci (*DDX39B, NFKBIL1, CDC42BPG, CDC63, BRAP, ACAD10*, and *PCNX3*) that might be novel susceptibility loci for hyperuricemia. However, five candidate SNVs at 12q24.1 exhibited moderate or strong LD (0.43 ≤ *r* ^*2*^ ≤ 0.99) with rs671 of *ALDH2* that has been reported to be associated with renal function-related traits (Levey et al. 2005; Sakiyama et al. 2016, 2017) (Online Resource 5). Thus, we did not consider the candidate SNVs at 12q24.1 as novel susceptibility loci for hyperuricemia. It has been reported that rs505802 of *SLC22A12* and rs504915 of *NRXN2* are related to the serum concentration of uric acid (Kolz et al. 2009; Okada et al. 2012). These SNVs are located relatively close (~ 240 kb) to rs55975541 of *CDC42BPG* at 11q13.1. However, these SNVs were not in LD (rs505802, *r* ^*2*^ = 0.06, *D*ʹ = 0.26; rs504915, *r* ^*2*^ = 0.10, *D*′ = 0.39) (Fig. 2). Therefore, it is possible that rs55975541 of *CDC42BPG* independently affects the serum concentration of uric acid. Consequently, four genes (*DDX39B*, *NFKBIL1*, *CDC42BPG*, and *PCNX3*) were identified as candidates for novel susceptibility loci that might be related to the serum concentration of uric acid. Of these candidates, the association of rs55975541 in *CDC42BPG* showed the significance level with *P* = 3.7 × 10^− 12^ in the dominant model (Online Resource 4).

### Replication studies for candidate SNVs related to CKD or hyperuricemia

We examined the relation of 17 candidate SNVs identified from our longitudinal EWASs to CKD or hyperuricemia using the Chi-square test or linear regression analysis, using cross-sectional data for CKD and hyperuricemia-related traits in 7699 Japanese subjects of the replication cohort (Online Resource 6). In this replication study, rs55975541 in *CDC42BPG* was significantly [*P* < 4.90 × 10^− 4^ (0.05/17 SNVs × 6)] related to the serum concentration of uric acid. We also assessed relations of the candidate SNVs detected in the discovery cohort with CKD or hyperuricemia, using GKDGEN meta-analysis data on European or African ancestry populations (Online Resource 7). The in silico replication study did not show significant association with the five candidate SNVs that were newly identified in the discovery cohort. However, four SNVs (rs2049330, rs2257212, rs1143671, and rs1143672) in *SLC15A2* were significantly (*P* < 4.90 × 10^− 4^) associated with eGFR in European ancestry populations, although these SNVs were related to the serum concentration of uric acid with borderline significance (*P* = 5.50 × 10^− 7^ to 6.40 × 10^− 7^) in our longitudinal EWASs.

### Survey of LD with disease-associated SNVs that were not examined in the longitudinal EWAS

We further investigated whether the allele frequency of the replicated target SNV in *CDC42BPG* was affected by other previously reported SNVs that were not included in the exome arrays used and which exhibited strong LD. LDproxy, an LDlink application, indicated rs55975541 in *CDC42BPG* was in LD (*r* ^2^ ≥ 0.8, *P* < 0.0001) with rs80094634 only in JPT (Japanese in Tokyo, Japan) from the 1000 Genomes Project database [http://www.internationalgenome.org, (The 1000 Genomes Project Consortium 2010)] (Online Resource 8). The nucleotide substitution at the non-genotyped SNV was silent (intronic variant), whereas the variant at the candidate SNV of *CDC42BPG* can alter an amino acid (nonsynonymous substitution). According to the Ensembl human database (http://www.ensembl.org/Homo_sapiens/ *)*, it is possible that the nonsynonymous substitution has an effect on the protein function because the amino acid substitution was predicted to be “probably damaging” with scores of zero by SIFT prediction and 0.998 by PolyPhen analysis. Therefore, rs55975541 is more likely to be related to hyperuricemia than, the non-genotyped SNV, rs80094634. The LDproxy analysis also showed that rs55975541 in *CDC42BPG* was not in LD with any genetic variants in the established *SLC22A11, SLC22A12*, and *NRXN2* loci (Online Resource 8).

### Relation of previously reported SNVs to renal function-related traits in longitudinal EWASs for the discovery cohort

We comprehensively assessed significance levels of 291 SNVs at 55 previously known CKD- or hyperuricemia-associated loci (Online Resource 9). Of the SNVs examined, 103 at 37 loci, including *ABCG2, GCKR*, and *UMOD*, were significantly (*P* < 3.39 × 10^− 7^) associated with renal function-related traits in any of the three genetic models. In the discovery cohort, MAFs of some previously reported SNVs were less than 5%. For instance, the SNV rs121907892 of *SLC22A12* was significantly associated with the prevalence of hyperuricemia and serum concentration of uric acid in all the genetic models (*P* < 2.0 × 10^− 16^). However, rs121907892 was excluded from our longitudinal EWAS because of low minor allele frequency (MAF < 0.03). The differences in significance levels between the present and previous studies might be due to sample sizes, statistical methods, or differences in genetic background among the populations examined.

## Discussion

In the present study, 17 SNVs showed significant relations with CKD- or hyperuricemia-related traits. Five of these SNVs have not been reported in previous studies. However, the relation of these SNVs to CKD or hyperuricemia could not be found in the replication study, except for rs55975541 in *CDC42BPG*. In the longitudinal EWAS for the discovery cohort, the SNV in *CDC42BPG* was significantly (*P* = 3.7 × 10^− 12^) associated with the serum concentration of uric acid. The association of rs55975541 was determined using cross-sectional data for the serum concentration of uric acid of 7699 Japanese subjects in the replication cohort. The LDproxy analysis suggests that this SNV independently affects the incidence of hyperuricemia.

MRCKγ encoded by *CDC42BPG* is ubiquitously expressed in a variety of tissues and organs including the kidney, according to The Human Protein Atlas database (http://www.proteinatlas.org/). This protein may act as a downstream effector of CDC42 in the regulation of cytoskeletal reorganization (Ng et al. 2004). The kidney-specific Cdc42 knockout mice exhibited early postnatal death due to renal failure (Choi et al. 2013). The nucleotide substitution at rs55975541 in *CDC42BPG* alters an amino acid residue at position 1237 (Arg1237Trp). The amino acid change may have an effect on the protein function, as predicted by SIFT and PolyPhen. Although *CDC42BPG* may be a susceptibility locus for renal function, the functional relevance of the candidate SNV to the serum concentration of uric acid remains unclear.

Despite many studies of associations between several *SLC* genes and hyperuricemia-related traits in diverse ethnic groups (Matsuo et al. 2008; Köttgen et al. 2013; Phipps-Green et al. 2016; Nakayama et al. 2017), association with *SLC15A2* has not been reported, according to the public databases. It is noteworthy that three nonsynonymous substitutions (at rs2257212, rs1143671, and rs1143672) in *SLC15A2* were significantly associated with eGFR in European ancestry populations from GKDGEN meta-analysis data, whereas these SNVs were related to the serum concentration of uric acid with a borderline significance in our longitudinal EWASs.

There were some limitations to the present study. First, the longitudinal EWAS was conducted in a local Japanese population only. Although we examined relations of candidate SNVs to CKD- or hyperuricemia-related traits in the replication studies, these studies were conducted in a cross-sectional manner. Therefore, replication of longitudinal EWASs in other Japanese populations or other ethnic groups is required to verify the relations of the identified SNVs to the relevant diseases. Second, the functional relevance of the candidate SNV to the pathogenesis of the diseases examined remains unclear. Therefore, further functional analysis is required to clarify the results of this study.

In conclusion, our findings indicated that rs55975541 in *CDC42BPG* was significantly associated with the serum concentration of uric acid in our EWASs. Genotyping for the SNV detected in our longitudinal EWAS may be informative for assessment of genetic risk for hyperuricemia.

## Electronic supplementary material

Below is the link to the electronic supplementary material.

Supplementary material 1 (PDF 222 KB)

Supplementary material 2 (PDF 228 KB)

Supplementary material 3 (PDF 222 KB)

Supplementary material 4 (PDF 206 KB)

Supplementary material 5 (PDF 208 KB)

Supplementary material 6 (PDF 242 KB)

Supplementary material 7 (XLSX 17 KB)

Supplementary material 8 (PDF 158 KB)

Supplementary material 9 (XLSX 93 KB)
