# Supplementary material for: Identification of CDC42BPG as a novel susceptibility locus for hyperuricemia in a Japanese population
Source: Mol Genet Genomics. 2017 Nov 9;293(2):371–9. doi: 10.1007/s00438-017-1394-1 (PMC5854719; doi:10.1007/s00438-017-1394-1)
Supplement: Supplementary file 3 — Supplementary material 3 (PDF 222 KB) [file 438_2017_1394_MOESM3_ESM.pdf]

Recessive model

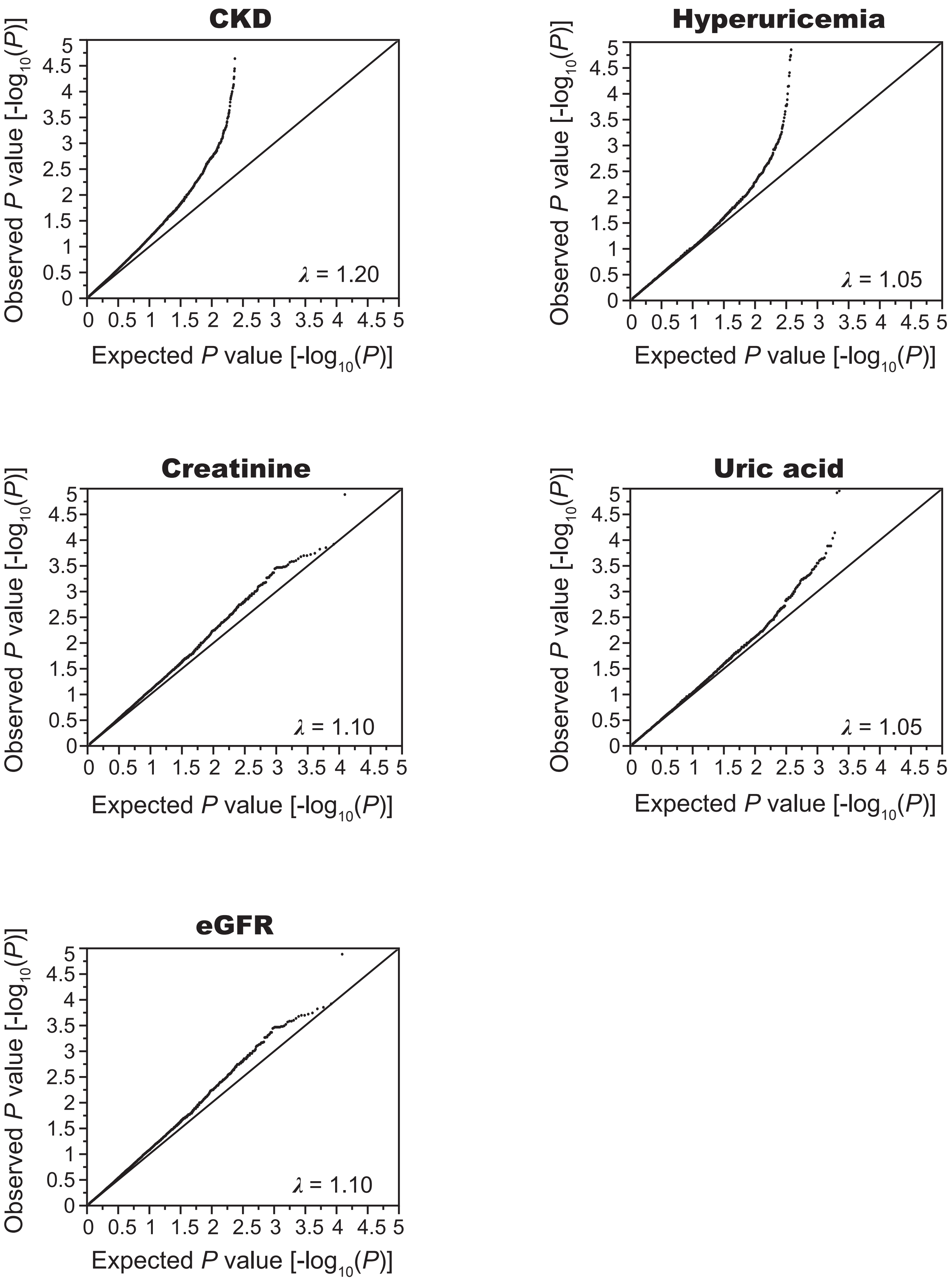

Figure S3. Quantile-quantile plots for *P* values in the longitudinal EWASs for the prevalence of CKD and hyperuricemia, eGFR, and serum concentrations of creatinine and uric acid in the recessive model. The observed *P* values (y-axis) were compared with the expected *P* values (x-axis) under the null hypothesis, with the values being plotted as $-\log_{10}(P)$. CKD, chronic kidney disease. eGFR, estimated glomerular filtration rate. λ represents the genomic inflation factor.
