# Supplementary material for: Identification of CDC42BPG as a novel susceptibility locus for hyperuricemia in a Japanese population
Source: Mol Genet Genomics. 2017 Nov 9;293(2):371–9. doi: 10.1007/s00438-017-1394-1 (PMC5854719; doi:10.1007/s00438-017-1394-1)
Supplement: Supplementary file 4 — Supplementary material 4 (PDF 206 KB) [file 438_2017_1394_MOESM4_ESM.pdf]

Table S1. Significant SNVs identified by the GEE model for the prevalence of CKD and hyperuricemia, serum concentrations of creatinine and uric acid, and eGFR in subjects of the discovery cohort, without dialysis patients and population outliers identified by the PCA method.

Genetic model	Trait/Disease	RefSNP ID	Location[a]	Alleles	Gene	Mutation	Estimate[b]	Std.err	Wald	P-value	MAF	Approxdf[c]
Dominant	Creatinine	rs11543349	20: 62,813,587	G→C	*OGFR*	Missense: G658R	0.02	0.003	32.5	1.2×10^{-8}	0.345	262
	Hyperuricemia	rs12229654	12: 110,976,657	T→G			-0.36	0.066	29.5	5.5×10^{-8}	0.263	244
		rs3782886	12: 111,672,685	T→C	*BRAP*	Synonymous: R241R	-0.38	0.065	34.3	4.9×10^{-9}	0.329	316
		rs11066015	12: 111,730,205	G→A	*ACAD10*	Silent	-0.40	0.065	37.2	1.1×10^{-9}	0.311	315
		rs671	12: 111,803,962	G→A	*ALDH2*	Missense: E504K	-0.38	0.065	34.7	3.8×10^{-9}	0.312	316
		rs2074356	12: 112,207,597	G→A	*HECTD4*	Silent	-0.38	0.065	34.6	4.1×10^{-9}	0.288	290
		rs11066280	12: 112,379,979	T→A	*HECTD4*	Silent	-0.36	0.065	31.3	2.2×10^{-8}	0.324	316
	Uric acid	rs3733591	4: 9,920,506	T→C	*SLC2A9*	Missense: R294H	10.19	1.748	34.0	5.5×10^{-9}	0.279	211
		rs13129697	4: 9,925,343	T→G	*SLC2A9*	Silent	-11.34	1.954	33.7	6.5×10^{-9}	0.482	212
		rs3775948	4: 9,993,558	C→G	*SLC2A9*	Silent	-12.67	1.867	46.1	1.2×10^{-11}	0.435	239
		rs2239709	6: 31,539,670	C→T	*DDX39B*	Silent	-9.51	1.828	27.1	2.0×10^{-7}	0.207	123
		rs2071593	6: 31,545,022	G→A	*NFKBIL1*	Silent	-9.49	1.830	26.9	2.2×10^{-7}	0.207	123
		rs505802	11: 64,589,600	C→T	*SLC22A12*		-17.32	1.978	76.7	$< 2.0\times10^{-16}$	0.188	101
		rs55975541	11: 64,829,729	G→A	*CDC42BPG*	Missense: R1237W	-13.79	1.986	48.3	3.7×10^{-12}	0.172	85
		rs10849915	12: 110,895,818	T→C	*CDC63*	Silent	-9.23	1.751	27.8	1.3×10^{-7}	0.265	185
		rs10774610	12: 110,902,439	T→C	*CDC63*	Silent	-9.19	1.751	27.6	1.5×10^{-7}	0.267	188
		rs12229654	12: 110,976,657	T→G			-10.07	1.754	33.0	9.3×10^{-9}	0.263	187
		rs3782886	12: 111,672,685	T→C	*BRAP*	Synonymous: R241R	-10.91	1.754	38.7	4.9×10^{-10}	0.329	248
		rs11066015	12: 111,730,205	G→A	*ACAD10*	Silent	-11.30	1.750	41.9	9.5×10^{-11}	0.311	249
Additive	Creatinine	rs11543349	20: 62,813,587	G→C	*OGFR*	Missense: G658R	0.01	0.003	30.9	2.7×10^{-8}	0.345	71
	eGFR	rs11543349	20: 62,813,587	G→C	*OGFR*	Missense: G658R	-1.32	0.251	27.4	1.6×10^{-7}	0.345	71
	Hyperuricemia	rs3775948	4: 9,993,558	C→G	*SLC2A9*	Silent	-0.03	0.005	27.0	2.0×10^{-7}	0.435	166
		rs12229654	12: 110,976,657	T→G			-0.04	0.006	38.9	4.5×10^{-10}	0.263	35
		rs3782886	12: 111,672,685	T→C	*BRAP*	Synonymous: R241R	-0.04	0.006	39.1	4.0×10^{-10}	0.329	69
		rs11066015	12: 111,730,205	G→A	*ACAD10*	Silent	-0.04	0.006	42.2	8.3×10^{-11}	0.311	57
		rs671	12: 111,803,962	G→A	*ALDH2*	Missense: E504K	-0.04	0.006	40.8	1.7×10^{-10}	0.312	57
		rs2074356	12: 112,207,597	G→A	*HECTD4*	Silent	-0.04	0.006	38.7	5.1×10^{-10}	0.288	47
		rs11066280	12: 112,379,979	T→A	*HECTD4*	Silent	-0.04	0.006	37.6	8.9×10^{-10}	0.324	67

Table S1 (continued)

Additive	Uric acid	rs3733591	4: 9,920,506	T→C	*SLC2A9*	Missense: R294H	9.29	1.370	45.8	1.3×10^{-11}	0.279	36
		rs13129697	4: 9,925,343	T→G	*SLC2A9*	Silent	-7.88	1.232	41.0	1.6×10^{-10}	0.482	189
		rs3775948	4: 9,993,558	C→G	*SLC2A9*	Silent	-10.62	1.243	73.0	$< 2.0\times10^{-16}$	0.435	126
		rs12801636	11: 65,623,846	A→G	*PCNX3*	Silent	6.47	1.235	27.4	1.7×10^{-7}	0.487	198
		rs3782886	12: 111,672,685	T→C	*BRAP*	Synonymous: R241R	-8.93	1.348	43.9	3.5×10^{-11}	0.329	53
		rs11066015	12: 111,730,205	G→A	*ACAD10*	Silent	-8.82	1.367	41.6	1.1×10^{-10}	0.311	43
		rs671	12: 111,803,962	G→A	*ALDH2*	Missense: E504K	-8.62	1.365	39.9	2.7×10^{-10}	0.312	43
		rs2074356	12: 112,207,597	G→A	*HECTD4*	Silent	-8.77	1.397	39.4	3.5×10^{-10}	0.288	36
		rs11066280	12: 112,379,979	T→A	*HECTD4*	Silent	-8.78	1.343	42.8	6.2×10^{-11}	0.324	53
Recessive	Uric acid	rs3782886	12: 111,672,685	T→C	*BRAP*	Synonymous: R241R	-0.01	0.002	37.0	1.2×10^{-9}	0.329	54
		rs11066015	12: 111,730,205	G→A	*ACAD10*	Silent	-0.01	0.002	40.0	2.6×10^{-10}	0.311	43
		rs671	12: 111,803,962	G→A	*ALDH2*	Missense: E504K	-0.01	0.002	40.0	2.5×10^{-10}	0.312	43
		rs2074356	12: 112,207,597	G→A	*HECTD4*	Silent	-0.01	0.002	38.1	6.8×10^{-10}	0.288	36
		rs11066280	12: 112,379,979	T→A	*HECTD4*	Silent	-0.01	0.002	35.1	3.1×10^{-9}	0.324	53

[a] Location in NCBI build GRCh38. [b] Estimate of coefficient. [c] A scale of small effective sample size: approxdf = 2 × MAF × Nindep, where Nindep is the sum of the estimated number of independent observations per person. Std.err, standard error. MAF, minor allele frequency.
