# Supplementary material for: Identification of CDC42BPG as a novel susceptibility locus for hyperuricemia in a Japanese population
Source: Mol Genet Genomics. 2017 Nov 9;293(2):371–9. doi: 10.1007/s00438-017-1394-1 (PMC5854719; doi:10.1007/s00438-017-1394-1)
Supplement: Supplementary file 5 — Supplementary material 5 (PDF 208 KB) [file 438_2017_1394_MOESM5_ESM.pdf]

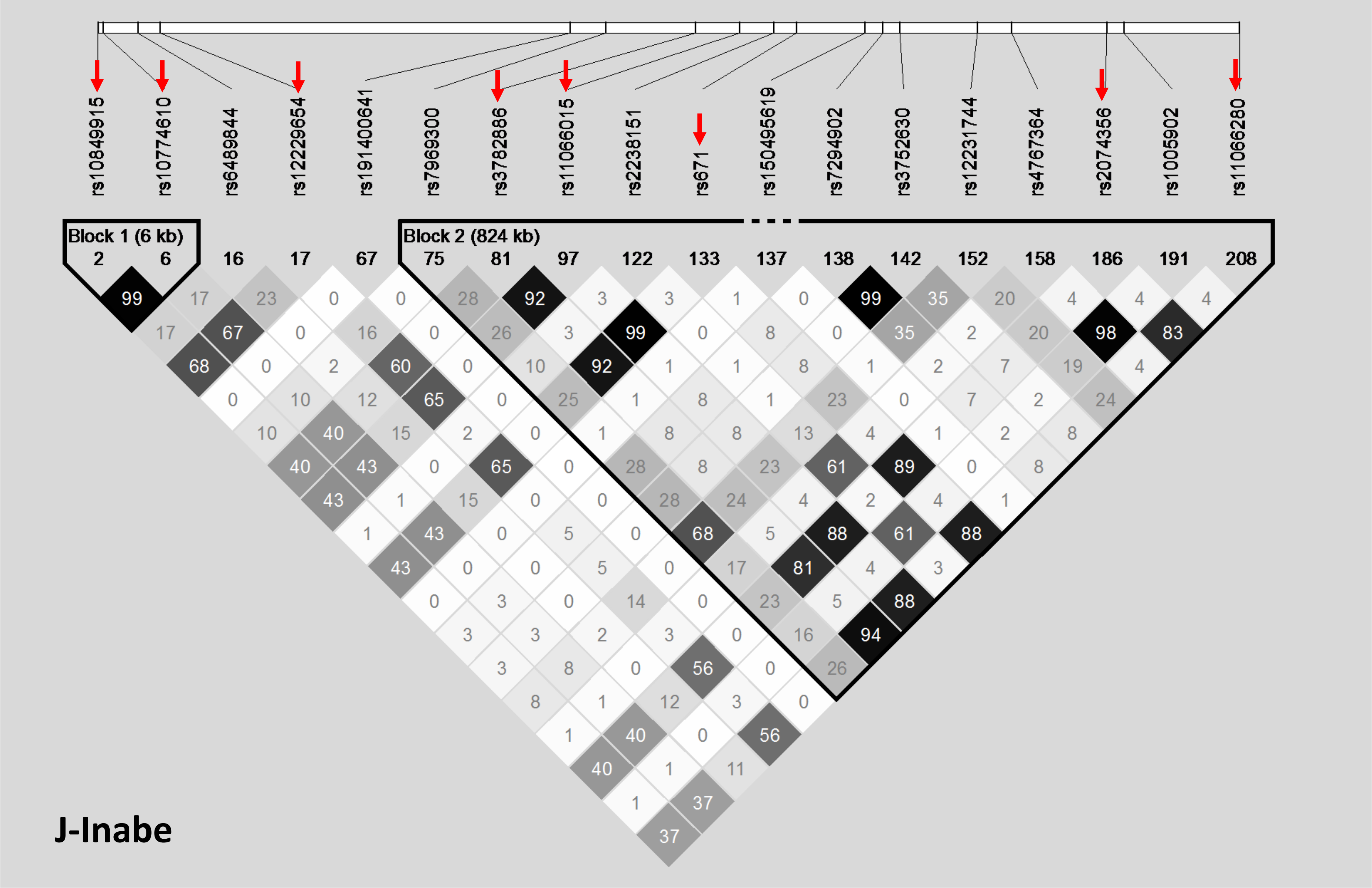

Figure S4. Linkage disequilibrium of 18 biallelic sites in a ~1.5 Mb genomic region at 12q24.1 based on SNV data for the discovery cohort. SNVs with minor allele frequency (MAF) of <0.01 were removed from the analysis. The positions of disease-associated SNVs are shown by the red arrows. JP-Inabe represents Japanese in Inabe city, Mie, Japan.
