# Supplementary material for: Identification of CDC42BPG as a novel susceptibility locus for hyperuricemia in a Japanese population
Source: Mol Genet Genomics. 2017 Nov 9;293(2):371–9. doi: 10.1007/s00438-017-1394-1 (PMC5854719; doi:10.1007/s00438-017-1394-1)
Supplement: Supplementary file 6 — Supplementary material 6 (PDF 242 KB) [file 438_2017_1394_MOESM6_ESM.pdf]

Table S2. Relation of candidate SNVs detected in the present study to cross-sectional data for renal function-related traits as determined by the chi-square test (for categorical data) or linear regression analysis (for quantitative data) in the discovery cohort.

RefSNP ID	Location[a]	Gene	Model	CKD (3402≤N≤3398)	Creatinine (6878≤N≤6885)	eGFR (6878≤N≤6885)	Hyperuricemia (5549≤N≤5556)	Uric acid (4301≤N≤4306)
**Novel association**								
rs2239709	6: 31,539,670	*DDX39B*	Additive	0.751	0.726	0.777	0.091	0.087
			Dominant	0.559	0.512	0.495	0.306	0.461
			Recessive	0.829	0.792	0.997	0.036	0.027
rs2071593	6: 31,545,022	*NFKBIL1*	Additive	0.751	0.726	0.777	0.091	0.087
			Dominant	0.559	0.512	0.495	0.306	0.461
			Recessive	0.829	0.792	0.997	0.036	0.027
rs55975541	11: 64,829,729	*CDC42BPG*	Additive	0.083	0.369	0.197	0.394	5.7×10^{-4}
			Dominant	0.174	0.843	0.650	0.264	**1.4×10^{-4}**
			Recessive	0.040	0.161	0.071	0.303	0.091
rs12801636	11: 65,623,846	*PCNX3*	Additive	0.528	0.342	0.891	0.682	0.210
			Dominant	0.275	0.230	0.837	0.775	0.115
			Recessive	0.894	0.698	0.632	0.395	0.200
rs11543349	20: 62,813,587	*OGFR*	Additive	0.190	0.254	0.081	0.017	0.095
			Dominant	0.581	0.315	0.408	0.030	0.245
			Recessive	0.167	0.360	0.089	0.015	0.035
**Novel association (but the allele frequencies are possibly correlated with previously reported SNVs)**								
rs10849915	12: 110,895,818	*CDC63*	Additive	0.005	9.3×10^{-4}	0.048	0.009	0.555
			Dominant	0.001	0.002	0.019	0.010	0.318
			Recessive	0.152	0.005	0.163	0.028	0.488
rs10774610	12: 110,902,439	*CDC63*	Additive	0.005	0.001	0.052	0.008	0.517
			Dominant	0.001	0.002	0.019	0.007	0.276
			Recessive	0.247	0.006	0.214	0.029	0.507
rs12229654	12: 110,976,657		Additive	0.004	**4.2×10^{-5}**	0.007	5.4×10^{-4}	0.345
			Dominant	9.5×10^{-4}	**1.0×10^{-5}**	0.002	0.006	0.426
			Recessive	0.184	0.040	0.285	7.8×10^{-4}	0.161
rs11066015	12: 111,730,205	*ACAD10*	Additive	0.001	**3.2×10^{-8}**	**2.4×10^{-4}**	0.003	0.360
			Dominant	**2.6×10^{-4}**	**4.5×10^{-8}**	**1.2×10^{-4}**	0.036	0.156
			Recessive	0.100	**1.6×10^{-4}**	0.011	0.002	0.521
rs11066280	12: 112,379,979	*HECTD4*	Additive	**3.6×10^{-4}**	**2.8×10^{-7}**	**1.5×10^{-4}**	0.001	0.399
			Dominant	**1.1×10^{-4}**	**1.0×10^{-7}**	**5.7×10^{-5}**	0.112	0.212
			Recessive	0.043	0.003	0.018	**2.6×10^{-4}**	0.369

Table S2 (continued)

**Previously reported association**								
rs3733591	4: 9,920,506	*SLC2A9*	Additive	0.254	0.419	0.081	0.103	0.018
			Dominant	0.118	0.190	0.053	0.040	0.005
			Recessive	1.000	0.804	0.635	0.848	0.200
rs13129697	4: 9,925,343	*SLC2A9*	Additive	0.731	0.898	0.799	0.503	0.165
			Dominant	0.534	0.659	0.624	0.417	0.592
			Recessive	0.554	0.776	0.552	0.306	0.058
rs3775948	4: 9,993,558	*SLC2A9*	Additive	0.723	0.675	0.887	0.168	0.019
			Dominant	0.648	0.376	0.760	0.176	0.131
			Recessive	0.686	0.737	0.795	0.101	0.006
rs505802	11: 64,589,600	*SLC22A12*	Additive	0.046	0.497	0.515	0.132	**8.5×10^{-10}**
			Dominant	0.016	0.329	0.426	0.079	**7.4×10^{-8}**
			Recessive	0.372	0.369	0.311	0.184	**9.4×10^{-7}**
rs3782886	12: 111,672,685	*BRAP*	Additive	0.001	**2.7×10^{-7}**	**2.6×10^{-4}**	0.001	0.253
			Dominant	**4.3×10^{-4}**	**2.5×10^{-7}**	**1.2×10^{-4}**	0.028	0.099
			Recessive	0.039	5.5×10^{-4}	0.012	0.001	0.497
rs671	12: 111,803,962	*ALDH2*	Additive	0.001	**1.2×10^{-8}**	**1.4×10^{-4}**	0.004	0.386
			Dominant	**1.9×10^{-4}**	**1.7×10^{-8}**	**7.4×10^{-5}**	0.047	0.176
			Recessive	0.073	**1.2×10^{-4}**	0.008	0.002	0.487
rs2074356	12: 112,207,597	*HECTD4*	Additive	0.002	**8.1×10^{-7}**	7.1×10^{-4}	0.004	0.444
			Dominant	6.0×10^{-4}	**5.3×10^{-7}**	**3.5×10^{-4}**	0.153	0.347
			Recessive	0.124	0.001	0.018	0.001	0.266

[a] location in NCBI build GRCh38. Based on Bonferroni's correction, *P*-values of $<4.90\times10^{-4}$ (0.05/17 SNVs × 6) were considered significant and are shown in bold. CKD, chronic kidney disease. eGFR, estimated glomerular filtration rate.
