# Supplementary material for: Identification of CDC42BPG as a novel susceptibility locus for hyperuricemia in a Japanese population
Source: Mol Genet Genomics. 2017 Nov 9;293(2):371–9. doi: 10.1007/s00438-017-1394-1 (PMC5854719; doi:10.1007/s00438-017-1394-1)
Supplement: Supplementary file 8 — Supplementary material 8 (PDF 158 KB) [file 438_2017_1394_MOESM8_ESM.pdf]

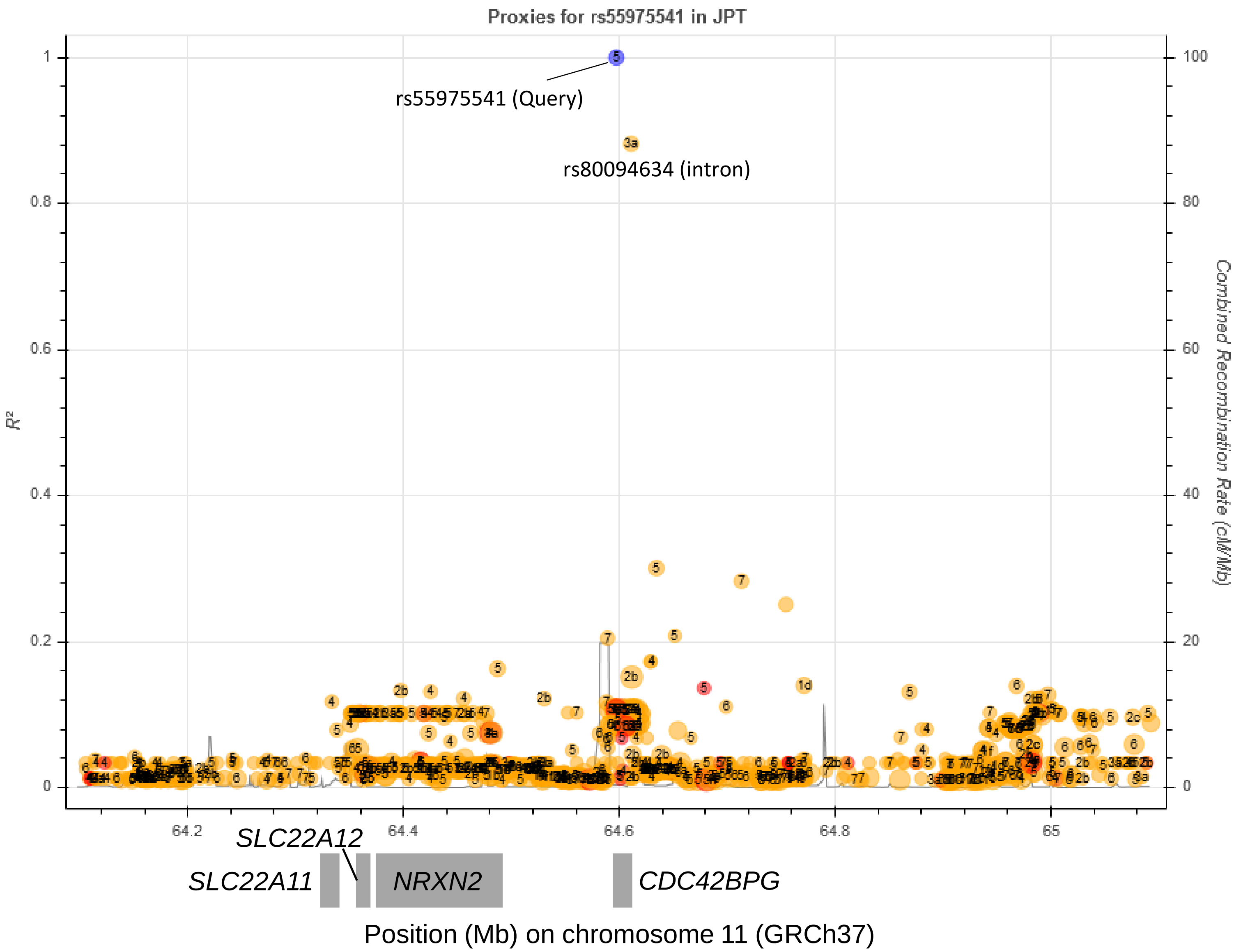

Figure S5. Correlations of rs55975541 with other SNVs around *CDC42BPG* using the LDproxy web-based application. The abscissa axis represents the chromosomal position (NCBI build GRCh37). The ordinate axis of circles represents an r^2 value with the query SNV. A red circle represents SNVs in a coding region. The size of the circle represents a minor allele frequency. The number in a circle represents a regulatory potential, as shown by the RegulomeDB score (http://www.regulomedb.org/, Boyle et al. 2012). The score decreases as the reliability of regulatory potential increases. The gray line indicates combined recombination rate (cM/Mb). The gray boxes indicate the chromosomal positions of *CDC42BPG*, *NRXN2*, *SLC22A11*, and *SLC22A12*.
